# Accuracy Evaluation of an Alternative Approach for a CAD-AM Mandibular Reconstruction with a Fibular Free Flap via a Novel Hybrid Roto-Translational and Surface Comparison Analysis

**DOI:** 10.3390/jcm12051938

**Published:** 2023-03-01

**Authors:** Mirko Bevini, Francesco Vitali, Francesco Ceccariglia, Giovanni Badiali, Achille Tarsitano

**Affiliations:** 1 IRCCS Azienda Ospedaliero Universitaria di Bologna, Oral and Maxillofacial Surgery Unit, Via Albertoni 15, 40138 Bologna, Italy; 2 Department of Biomedical and Neuromotor Sciences, University of Bologna, 40138 Bologna, Italy

**Keywords:** mandibular reconstruction, Maxillofacial Surgery, analysis method, fibular flap

## Abstract

Although the fibula free flap represents the gold standard for mandibular reconstructions, when implanted as a single barrel, this flap does not have the cross-sectional requisites to restore the native mandibular height, which is in turn required for the implant-supported dental rehabilitation of the patient. Our team has developed a design workflow that already considers the predicted dental rehabilitation, positioning the fibular free flap in the correct craniocaudal position to restore the native alveolar crest. The remaining height gap along the inferior mandibular margin is then filled by a patient-specific implant. The aim of this study is to evaluate the accuracy in transferring the planned mandibular anatomy resulting from said workflow on 10 patients by means of a new rigid body analysis method, derived from the evaluation of orthognathic surgery procedures. The analysis method has proved to be reliable and reproducible, and the results obtained show that the procedure already has satisfactory accuracy (4.6° mean total angular discrepancy, 2.7 mm total translational discrepancy, 1.04 mm mean neo-alveolar crest surface deviation), while also pointing out possible improvements to the virtual planning workflow.

## 1. Introduction

In the last two decades, CAD-CAM technologies, mainly through the advances in additive manufacturing (AM), have been successfully applied to mandibular reconstructions using autologous transplanted bony or chimeric flaps after mandibular resection [1,2]. Virtual surgical planning has evolved from planning the procedure in silico and transferring it to the patient via cutting guides while also modeling stock plates onto rapid-prototyped patient anatomical models, to replacing the stock titanium reconstructive plates with custom-manufactured patient-specific implants (PSIs). The computer-assisted surgical approach has already proved reliable and capable of reducing surgical times, patients’ hospital stays, and overall recovery, while also providing improvements in functional and aesthetic outcomes [3,4]. PSIs have also proved superior in terms of their mechanical strength, reducing the risk of re-intervention for plate replacement [5].

The design process for mandibular reconstructive PSIs, however, has remained largely unvaried throughout its brief history, mimicking the shape and function of hand-modeled stock titanium plates [6]. The guiding aspect of the procedure is the inferior mandibular edge, which is replaced with fibular or iliac crest free flaps, connected to the remaining mandibular segments via the PSI until ossification of the osteotomy interfaces is achieved. The said flaps, however, lack the cross-sectional shape requirements needed to reinstate the height of the native mandible, which in turn leads to well-known complications tied to the placement of dental implants onto bony flaps, as part of a comprehensive masticatory rehabilitation process [7,8,9].

The current proposed solutions include using an iliac crest flap, which is only useful for shorter bony gaps [10]; using a ‘double-barrel’ configuration of the fibular flap, which again shortens the bridgeable mandibular gap and increases the complexity of the procedure [11,12]; and using vertical distraction osteogenesis on the basally positioned fibular flap, which causes discomfort to the patient and is technically complex, increasing the risks of infection and fracture [13,14].

**Figure 1:**
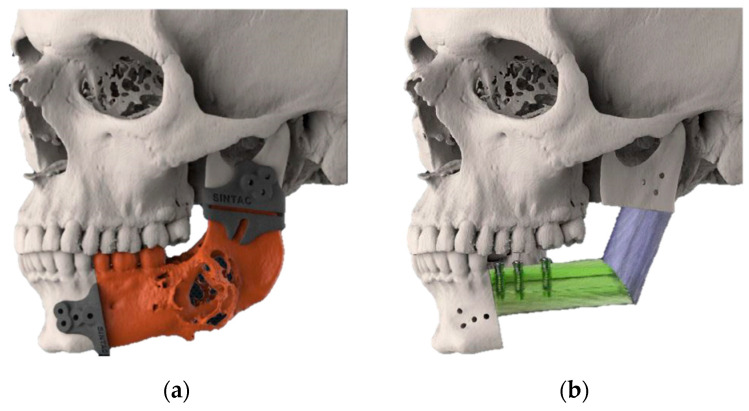
(**a**) Lateral view of resection plans defined by the tumor extent. (**b**) Lateral view of mandibular reconstruction planning with two fibula segments. In the mesial segment, dental implants were placed.

**Figure 2:**
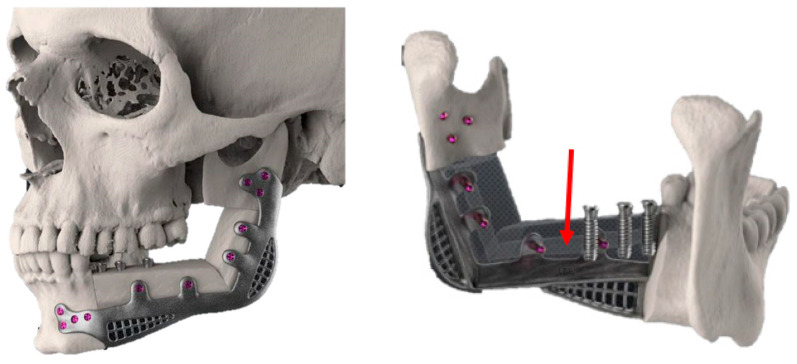
Titanium plate planning. The PSI features an accommodation for the placement of fibula segments (shown by red arrow). A grid is present for better integration with soft tissue.

Our team developed a design workflow for the reconstructive procedure that already considers the predicted dental rehabilitation, positioning the segments of a fibular free flap in a craniocaudal position aimed at reconstructing the native alveolar crest. The use of standard dental implants is then possible, theoretically reducing complications tied to incorrect biomechanical loads (Figure 1). The remaining height gap along the inferior mandibular margin is then filled by a specifically designed portion of the PSI used to keep the fibular segments in the planned position (Figure 2). This design concept was first proposed by our group in 2021 [15].

In doing so, the reconstructive virtual surgical plan (VSP) must be transferred to the patient with the highest possible accuracy in order to keep the subsequent implant surgery and dental prosthetics design as unaltered as possible. The clinical results pertaining to this trial were recently published by our team [16].

The present study aims at validating the proposed workflow on 10 patients by means of a novel analysis technique, based on methods used for an orthognathic surgery outcome analysis, which was used to assess the reconstructive surgical outcomes in terms of the planned mandibular anatomy transfer precision.

This analysis method outputs a more clearly interpretable overview of the outcome discrepancies from planning when compared with the more widely used surface comparison methods, allowing clinicians and technicians to improve the design and surgical workflow to address critical aspects.

## 2. Materials and Methods

**Scheme 1:**
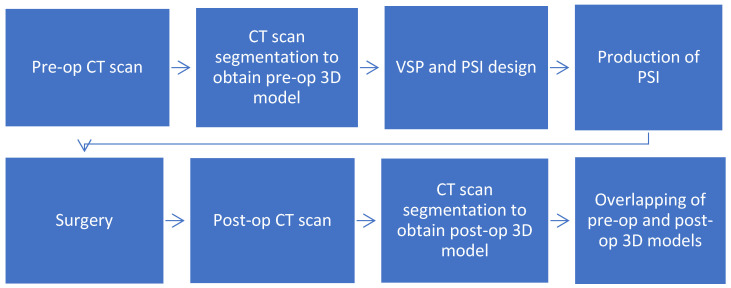
Workflow diagram.

The workflow used in this study is explained in Scheme 1.

### 2.1. Patients Population

Eleven patients were prospectively enrolled in the study (Table 1) (10 male, 1 female, mean age of 41.5 years), suffering from benign and low-grade early-stage malignant diseases requiring mandibular resection. All patients underwent primary surgical mandibular resection and reconstruction with a fibula free flap between January 2019 and May 2021, at the Maxillofacial Surgery Unit of IRCCS Azienda Ospedaliera di Bologna University Hospital. Written informed consent to the procedure and data publication was acquired from all patients. One patient was not included in the present analysis due to follow-up drop-out.

### 2.2. Virtual Surgical Planning and PSI Design

For the preoperative planning and design, DICOM data from patients’ CT scans of the head and neck and CT angiography of the legs, together with virtual dental casts in .STL format, were processed in the Materialise Mimics, Materialise 3-Matic (Materialise, Leuven, Belgium), and Geomagic Freeform-Plus software programs (3D Systems, Rock Hill, SC, USA) to obtain a virtual 3D model of the patient’s facial anatomy, to plan the resection, and to plan the position of the bony segments of the fibular free flap. VSP for dental implant placements according to the projected dental prosthetics, based on the virtual dental casts, was also performed (Figure 1).

The custom-made reconstructive PSI was designed following the ideal lateral and caudal mandibular surface, generated via mirroring or the superimposition and scaling of healthy mandibular models, making use of a lattice design to reduce the overall volume and provide anchorage for the oral floor muscles. The cranial segment of the plate was designed to support the fibular flap in the planned position, comparable with the native alveolar crest (Figure 2). Mandibular cutting templates and osteotomy guides for the free fibular bone flap were designed based on the VSP in 3-Matic software.

The PSI was manufactured via DMLS from Ti6AlV4 powder using an EOSINT M280 system (Electro-Optical Systems GmbH, Munich, Germany). The PSI has a minimum thickness of 2 mm and is fixed to the native mandible and fibular segments with 2.3 mm screws. PSI sterilization was carried out via an autoclave cycle at 121 °C for at least 15 min.

### 2.3. Surgery

All patients underwent a mandibular reconstruction using a fibular free flap.

After mandibular exposure, cutting guides are temporarily fixed in the planned position via screws. The mandibular surgical resection is then performed according to VSP. Cutting guides also act as drilling guides for PSI positioning and screw fixation. 

**Figure 3:**
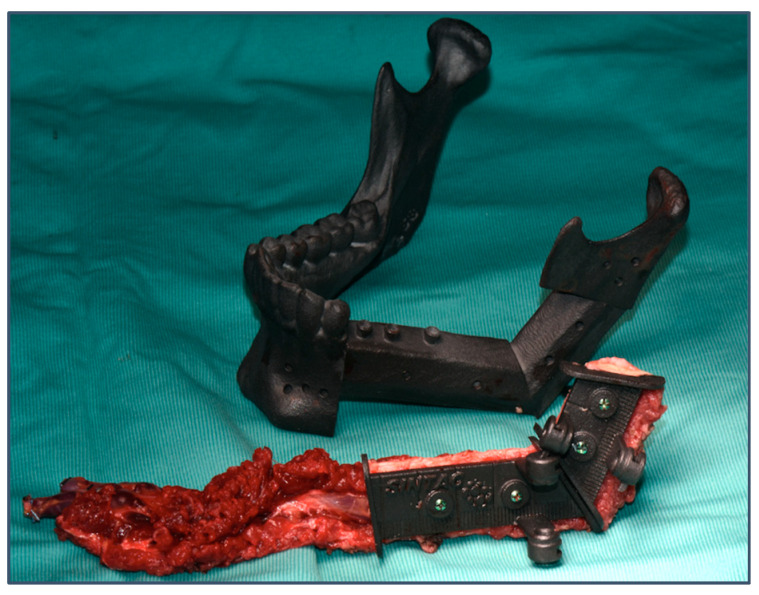
Free fibula flap consisting of two bony segments. The flap is compared morphologically with the 3D model of the mandibular reconstruction.

The fibular flap segments are cut under the guide of a custom cutting template fit onto the flap and temporarily anchored via screws (Figure 3).

**Figure 4:**
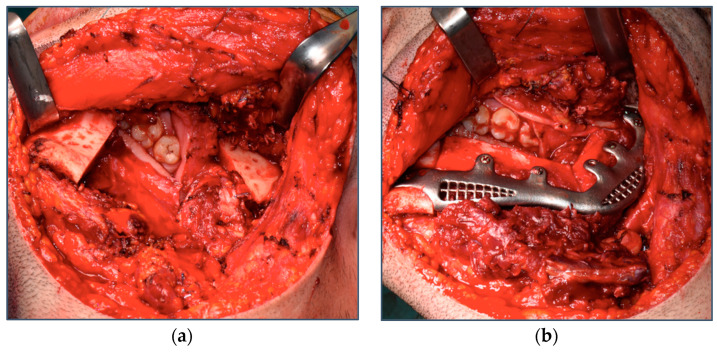
Intraoperative images of (**a**) a bone defect from mandibular resection and (**b**) free fibula flap on a custom-made reconstructive plate in a definitive position.

Segments are then assembled onto the PSI in the planned shape and fixed via 2.3 mm screws. The reconstructive ensemble is then placed in its planned position using the screw holes previously drilled into the mandibular segments (Figure 4).

### 2.4. Data Analysis

One to three months post-operation, all patients underwent a CT scan to assess the outcome of the procedure as well as to evaluate the osteotomy interface ossification and confirm the previously planned dental implant placement.

The DICOM data from each post-operative CT scan were segmented in Materialise Mimics software (Version 21.0) to obtain 3D models of the PSI, the native mandibular segments, and the transplanted fibular bone.

The evaluation of the accuracy of the bone congruence and planned neo-mandible anatomy between the VSP and post-operative outcome was performed using CloudCompare software (Version 2.1).

#### 2.4.1. Overlapping Procedure and Roto-Translational Discrepancy Computation

To evaluate the accuracy of the post-operative result, the planned and post-operative 3D models of the neo-mandible and plate in STL format were loaded onto the CloudCompare software program.

A common frame of reference is set by aligning planned and post-operative PSI 3D models via point pair alignment, which are then refined via iterative closest point (ICP) alignment. This alignment is subsequently checked via the generation of a colorimetric surface map to avoid misalignments due to segmentation artifacts. The bony segments are then aligned according to the position of the PSI models.

To better evaluate the single components of the neo-mandible, in the postoperative model the portions of the native mandible are then analyzed as independent entities (considered mandibular segments, CMSs), while the microvascular flap is considered as a further entity.

**Figure 5:**
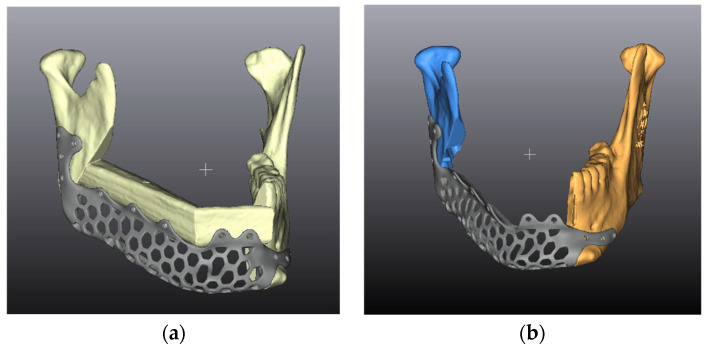
(**a**) Example of a 3D model of reconstructive planning. (**b**) Example of the obtained CMSs. CMSmaj is shown in orange, CMSmin in blue.

For each patient, except for cases needing resections including the condyle, upon the mandibular resection two mandibular portions are obtained: one greater (CMSmaj) and one smaller (CMSmin) (Figure 5b). The segments acquire different properties due to the different contact surfaces with the PSI, the presence of teeth, and the screw fixation to the PSI.

An ICP alignment of the analogous planned and post-operative CMSs is then performed. The roto-translation of this alignment identifies the cause of unwanted movement of the CMS with respect to the plan and the inaccuracy of its position (Figure 5).

The displacement is represented according to Euler’s angles convention (pitch, roll, and yaw angles; craniocaudal, antero-posterior, and lateral translations). A medio-lateral convention is then applied to the obtained angles and translations.

#### 2.4.2. Rotation Angles

**Figure 6:**
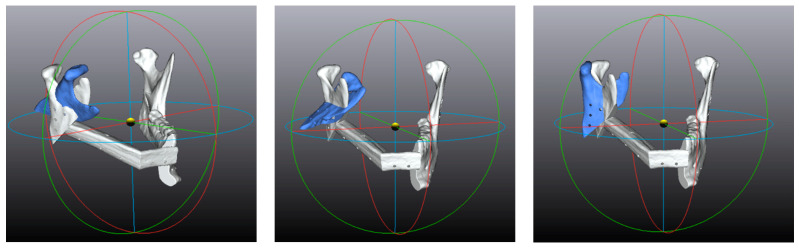
Examples of translation angles obtained with arbitrary values (50°): from **left** to **right**, pitch, roll, and yaw.

The rotation angles were tabulated according to the following convention (Figure 6):

- A positive pitch angle indicates a clockwise rotation in a right lateral projection;
- A positive roll angle indicates a lateral displacement of the caudal part in an antero-posterior projection;
- A positive yaw angle indicates lateral displacement of the posterior part in a craniocaudal projection.

#### 2.4.3. Translation Vector

**Figure 7:**
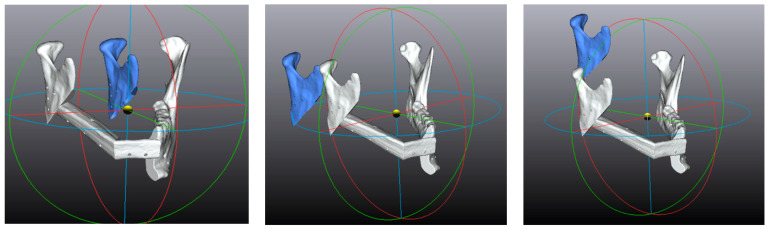
Examples of translation vectors obtained with arbitrary values (50 mm): from **left** to **right**, X, Y, and Z.

The translation vectors are considered as X, Y, and Z components (Figure 7):

- A positive X component indicates a lateral displacement;
- A positive Y component indicates a posterior displacement;
- A positive Z component indicates a cranial displacement.

#### 2.4.4. Total Discrepancies

To avoid negative and positive values canceling each other on average, the total rotation of each element is defined as the angle in the axis–angle conventional representation of rigid body transformation. This value is always positive, since the orientation of the rotation is defined by the direction of the axis around which it is performed. This rotation of the 3D model can be interpreted as the total angular discrepancy between the obtained position and the preoperative planning. 

Similarly, the total translational discrepancy is defined as the module of the translation vector calculated from its X, Y, and Z components (Table 2 and Table 3).

The data were divided by CMS segment size (CMSmaj or CMSmin) and tabulated accordingly. The descriptive statistics were computed for each group (Table 4).

#### 2.4.5. Evaluation of the Fibula Flap Placement

To verify the fibular positioning at a height compatible with the reconstruction of the alveolar crest, the most cranial segment of the fibula flap (fibular cranial segment, FCS) in the post-operative model (FCSpost-op) and in the planned model (FCSplan) are isolated.

**Figure 8:**
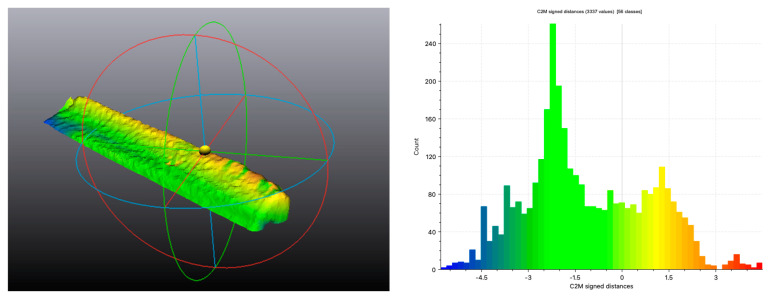
Surface overlap between FCSpost-op and FCSplan and colorimetric map obtained from the surface comparison. The X-axis shows the signed discrepancy in mm, while the Y-axis shows the number of points.

A closest-point comparison is then performed between the two segments, visually represented by a colorimetric map (Figure 8; Table 5).

The mean distance and standard deviation of the FCSpost-op points relative to the planning were also computed.

The distance data show positive values if the FCSpost-op is more cranial than planned, and negative values if it is more caudal.

The above data were also analyzed in terms of absolute values to obtain an overall average representative of the positioning discrepancy expressed in millimeters.

## 3. Results

Intraoperatively, the procedure was successful in all patients. One patient experienced late flap necrosis and concomitant intraoral plate exposure, due to which the PSI was replaced with a bridging plate. Subsequent dental implant positioning and prosthetic rehabilitation have been completed on 5 patients to date.

Table 2 shows all values obtained from the analysis of the CMS roto-translations. 

The descriptive statistics of the considered data are displayed in Table 3 and Table 4.

**Table 2:** Values obtained for each patient from the analysis of the CMS roto-translation process. Right-side segments are highlighted in green, left-side segments in yellow.

Patient	Yaw	Roll	Pitch	X Tran	Y Tran	Z Tran	Total Angle	Vector Tran
		deg			mm		deg	mm
P1 R	−2.2870	−1.0332	−0.6487	−0.6380	0.8925	1.1069	2.5969	1.5585
P1 L	2.6565	1.8258	5.7234	0.4159	−4.2463	4.5701	6.5307	6.2522
P2 R	−0.8278	2.8698	−1.2407	2.0277	1.4660	1.1429	3.2262	2.7508
P3 R	9.5146	2.6141	1.0442	0.2198	1.0542	0.0092	9.8984	1.0769
P3 L	−5.6032	−2.3560	−2.6749	0.1802	3.7489	−3.3179	6.6863	5.0095
P4 R	6.8338	−1.7266	−2.3379	−0.7012	1.1391	2.5669	7.3924	2.8945
P4 L	2.5102	3.1670	0.2459	1.5999	−1.5890	2.8016	4.0437	3.5964
P5 R	−4.7002	−1.7538	3.5939	−1.2017	0.7079	0.8815	6.1284	1.6499
P5 L	0.5635	−1.5256	0.8751	−1.0242	−0.4140	0.4095	1.8503	1.1782
P6 R	0.6577	−1.6379	2.4205	0.6853	−0.1187	−1.0178	3.0031	1.2327
P6 L	2.6078	−1.1542	−3.8083	−0.5921	0.3365	0.6444	4.7362	0.9376
P7 R	1.4263	2.1854	−0.1211	0.1192	0.6439	0.5008	2.6131	0.8244
P7 L	−1.1826	2.2436	0.7149	0.5981	1.5212	−0.1115	2.6410	1.6384
P8 R	1.3528	−0.2429	0.3496	−0.2516	0.0147	0.0111	1.4186	0.2523
P8 L	−1.1454	2.3242	1.6231	0.6690	0.3668	2.9990	3.0686	3.0945
P9 R	−0.1308	4.1515	1.4995	2.7059	−0.1459	3.3774	4.4170	4.3301
P9 L	−7.5972	1.0202	1.8874	−0.0182	0.0023	−0.0052	7.9100	0.0191
P10 R	0.0939	2.2603	5.3620	−3.8961	2.9153	8.4539	5.8179	9.7543
P10 L	−3.4093	0.5100	−0.5153	−0.4747	−1.7103	1.6226	3.4827	2.4048

**Table 3:** Descriptive statistics of the overall roto-translational results of CMSs.

	Yaw	Roll	Pitch	X Tran	Y Tran	Z Tran	Total Angle	Vector Tran
		deg			mm		deg	mm
Mean	0.0702	0.7232	0.7841	0.0223	0.3466	1.4024	4.6032	2.6555
Median	0.0939	1.0202	0.7950	0.1192	0.3668	0.8815	4.0437	1.6499
StDev	4.0372	2.0606	2.5387	1.3892	1.7125	2.4569	2.3166	2.3788
IQR	3.7030	3.6322	2.3373	1.2486	1.2289	2.6741	3.5075	2.2179

**Table 4:** Descriptive statistics regarding the roto-translation of the segments divided by CMS segment size (CMSmin—grey; CMSmaj—pink).

	Yaw	Roll	Pitch	X Tran	Y Tran	Z Tran	Total Angle	Vector Tran
		deg			mm		deg	mm
Mean	0.5151	0.2475	0.6841	−0.7201	0.9537	1.5064	5.3933	2.1980
Median	0.0939	−0.2429	0.7149	−0.5921	0.8925	0.6444	5.8179	1.5585
StDev	5.3609	1.8021	2.8092	1.3075	0.8977	2.7401	2.8056	2.9577
IQR	4.8947	3.3979	2.5362	0.6829	0.8026	1.0978	4.7514	0.7123
Mean	−0.3302	1.1514	0.8841	0.6904	−0.1999	1.3088	3.8922	3.0674
Median	0.2163	2.0056	0.8751	0.5424	−0.1323	1.3827	3.3545	2.9227
StDev	2.5838	2.2749	2.4044	1.1371	2.1093	2.3192	1.5970	1.7731
IQR	2.3001	3.7501	2.0090	1.2368	1.8699	2.5173	1.3042	2.6210

The overall discrepancies are below 5° in rotation and below 3 mm in translation, with the widest varying rotation being the yaw (4° StDev) and the widest varying translation being in the craniocaudal axis (2.5 mm).

In the analyzed sample, the average total angular discrepancy of the CMSmin segments is greater than that of the CMSmaj segments (5.4° vs. 3.9°), with the widest varying components being the yaw in both subsamples.

No specific positional tendency stands out in the signed average values, meaning that the variations fluctuate around the planned position.

Regarding the average translational error, CMSmaj exhibits a marginally greater value when compared to CMSmin (3.1 mm vs. 2.2 mm); however, this result is likely a byproduct of the analysis method. 

Table 5 shows the data obtained through the overlap between FCSplan and FCSpost-op. 

Analyzing the average of the mean distance in terms of the absolute value, the surface deviation between FCSplan and FCSpost-op is 1.04 mm, while the average signed distance is −0.28 mm, showing a tendency to precisely position the fibular segment around the planned position.

**Table 5:** FCS overlap data for each patient.

	Mean Distance	StDev	Absolute Value Mean Distance
		mm	
P1	1.0393	2.0781	1.0393
P2	−1.5001	1.6797	1.5001
P3	2.7833	1.9949	2.7833
P4	−0.5829	1.3361	0.5829
P5	−0.4723	1.0042	0.4723
P6	−1.1455	1.9461	1.1455
P7	−1.1102	2.2934	1.1102
P8	−0.0108	2.2794	0.0108
P9	−0.2937	0.8400	0.2937
P10	−1.4932	1.9029	1.4932
Mean	−0.2786	1.7355	1.0431
Median	−0.5276	1.9245	1.0747
StDev	1.3218	0.5124	0.7902
IQR	1.0552	0.6353	0.9063

## 4. Discussion

To date, the fibular free flap has become the gold standard in mandibular reconstruction. The current literature shows how the application of CAD/CAM technology to a mandibular reconstruction with a free fibula flap can improve the surgical outcomes both from functional and aesthetic points of view over traditional techniques [2,3], along with reductions in post-operative morbidity and surgical time [17].

The cross-sectional discrepancy between the native mandible and the fibula, however, is still an issue to be completely addressed. Traditionally, fibular segments are placed along the lower edge of the native mandible, supporting biomechanical loads in a similar way to the native mandible itself, while also obtaining a natural-looking mandibular contour. This technique, however, results in a neo-mandible that is approximately half the height of the native mandible [8].

Such discrepancies require complex dental prosthetics to compensate for incorrect intermaxillary relationships. This predisposes the patient to unbalanced dental biomechanical loads and peri-implantitis, and results in lower implant survival and success rates when compared to implants placed on the mandibular bone [7,8,9]. 

The proposed solutions mainly include doubling the fibular barrels over themselves, the use of vertical distractions of the fibular segment, or the use of free fibular grafts along the vascularized transplant. All three of these solutions increase the risk of complications such as flap failures, infections, or neo-mandibular fractures [11,12,13,14,18,19,20].

The approach we proposed is based on the positioning of the fibular flap at the same height of the native alveolar crest while recreating a natural-looking mandibular profile using a single PSI, which also stabilizes the fibular segments. This minimizes the gap between the bone flap and the native alveolar crest, reducing the risks of complications during implant rehabilitation, while at the same time restoring the patient’s mandibular profile [7,16].

Consequently, the comprehensive reconstructive planning process, which already considers the dental implant positions and prosthetics, needs to be transferred into practice in the patient with great accuracy. The main unpredictable factors to be accounted for in this approach are peri-fibular soft tissues, the exact section of the fibula in the used portion, together with the condyle–fossa relationship, which may vary as a consequence of surgery. 

The most widely used method in the current literature to evaluate the accuracy of VSP transfer to the patient in mandibular reconstructions is based on the superimposition of planned and post-operative 3D models via a closest-point registration algorithm followed by computing the closest point deviation between the two [6,21,22]. Used in a complex shape, however, the results output by similar analyses can be hard to interpret, if not misleading [23]. Subsequently, their use in improving the procedures they are aimed at analyzing is limited, as they do not express parameters that can be addressed during surgery. 

Therefore, to evaluate the planning transfer accuracy, we developed a new method based on the OrthoGnathic Analyzer 2.0, which is used for the evaluation of the planning transfer process in orthognathic surgery [24], and which we further modified to be used in mandibular reconstruction cases. 

The method is still based on the superimposition of the post-operative result with the virtual surgical planning, but considers single bony segments as rigid bodies, expressing the discrepancies in terms of translational and rotational movements along the three axes of space. The method also makes it possible to evaluate the positioning along the craniocaudal axis of the fibula free flap, subsequently adjusting the correct positions of future dental implants, further minimizing the risk of failure and improving the overall dental restoration. 

In the present analysis, the surface comparison was still used, although it was limited to the evaluation of the craniocaudal position of the neo-alveolar crest, obtaining useful clinical information as opposed to the comparison of the whole 3D mandibular model. The analysis of the fibular position is the most unpredictable factor of the whole procedure, as the cross-section of the fibula varies throughout its length and the exact section used for the vascularized transplant is not precisely predictable. A rigid body analysis is not viable in such scenarios.

In the analyzed sample, the average total angular discrepancy of the CMSmin segments is greater than that of the CMSmaj segments, thereby contributing the most to the overall modest postoperative discrepancy. It can be hypothesized that the reduced contact surface available between the bony segment and PSI, together with the unbalanced muscular and soft tissue connections, may explain this tendency. Another factor that may be contributing is the remaining dental occlusion between the maxilla and CMSmaj, which may serve intraoperatively as a guide and post-operatively as a stabilizing feature. The main rotation of the CMSmin segment is around the craniocaudal axis, as per a rotation of the condyle in the glenoid fossa. The translations are overall negligible. In addition, there is no specific positional tendency in any segment, as the rotations and translations fluctuate around the planned position (mean values close to zero).

These data must be taken as a starting point to implement solutions to limit CMSmin rotation and further improve a technique that already offers excellent accuracy, confirming previous published studies about CAD/CAM-guided mandibular reconstructions [2,3]. The accuracy is close to what was obtained in orthognathic procedures analyzed using a slight variation of this method [24].

CMSmaj exhibits a marginally greater total translational discrepancy when compared to CMSmin; however, this result is likely a byproduct of the analysis method. 

In terms of alveolar crest reconstructions, the fibular bone neo-alveolar crest deviates a negligible 1.04 mm from the native alveolar crest position, thereby eliminating the discrepancy present after reconstruction with the traditional “single barrel” technique. This discrepancy is analogous, if not closer to the original mandible, than what is obtained with a double barrel fibula or iliac crest graft, without the drawback of only being able to fill mandibular gaps shorter than 10 cm [20].

The average signed distance of −0.28 mm highlights the flap placement, which does not show a tendency for predominantly superior or inferior displacement. With the average signed distance being close to zero, the discrepancy fluctuates around the ideal position. 

The main limitation of this study is the small sample size, which will be increased with further applications of this workflow. Regarding the workflow itself, the possible plate-induced scattering and risk of plate exposure limit the application of this approach to patients not requiring radiotherapy, such as patients affected by benign or early-stage low-grade malignant tumors.

These preliminary results, however, are encouraging for the further expansion of the patient population and improvement of the technique itself, together with the broader application of the analysis technique to other hard tissue reconstruction procedures.

## 5. Conclusions

In conclusion, the analysis method introduced here proved to be reliable and reproducible in mandibular reconstruction cases, pointing out key aspects to be improved in the virtual surgical planning workflow. These aspects are mainly tied to the control of the smaller and non-teeth-bearing segment of the mandible, as well as focusing on the harvest of a specific section of the fibular bone so as to obtain the most accurate outcome possible when transferring the procedure to the patient in the theatre.

## Figures and Tables

**Table 1:** The patient population.

	Sex	Age	Pathology	N° Fibula Segments	N° Dental Implants
1	M	52	Ameloblastoma	2	/
2	M	43	Ameloblastoma	2	4
3	M	44	Ameloblastoma	2	/
4	M	25	Osteomyelitis	2	/
5	M	30	Ameloblastoma	2	3
6	F	37	Ameloblastoma	2	3
7	M	61	Osteomyelitis	1	/
8	M	56	Glandular odontogenic cyst	2	4
9	M	39	Osteosarcoma	2	/
10	M	27	Odontogenic keratocyst	1	/
11	M (excluded)	42	Verrucous carcinoma	2	/
